# Severe thyroiditis induced by sintilimab monotherapy in a patient with non-small cell lung cancer: a case report and literature review

**DOI:** 10.3389/fimmu.2025.1548452

**Published:** 2025-02-25

**Authors:** Xiaolin Zhao, Xiaoyu Wang, Surui Liu, Pian Cheng, Jinjuan Chen, Jie Liu

**Affiliations:** ^1^ School of Clinical Medicine, Shandong Second Medical University, Weifang, China; ^2^ Department of Oncology, Central Hospital Affiliated to Shandong First Medical University, Jinan, China

**Keywords:** sintilimab, irAEs, thyroiditis, NSCLC, case report

## Abstract

Thyroid dysfunction is a common immune-related adverse event (irAE) associated with immune checkpoint inhibitors (ICIs) that target PD-1, PD-L1, and CTLA-4. Nevertheless, the incidence of severe cases, defined as grade 3 or higher, remains rare. This report presents a detailed case study of severe thyroiditis in a patient with non-small cell lung cancer (NSCLC) who developed grade 3 thyroiditis following a single cycle of sintilimab monotherapy. The clinical presentation in this patient was remarkable for its early onset, occurring one week after the initiation of sintilimab therapy, and for its severe manifestations. During hospitalization, a prompt and accurate differential diagnosis was performed. Sintilimab treatment was discontinued, and the patient was promptly started on high-dose glucocorticoids, with a tapering schedule implemented as the condition improved or reached Common Terminology Criteria for Adverse Events (CTCAE) grade 1 or lower. The patient subsequently developed overt hypothyroidism, necessitating the initiation of thyroxine replacement therapy. Furthermore, we provide a comprehensive review of the mechanisms and risk factors associated with thyroid dysfunction immune-related adverse events (TD-irAEs). It is imperative for clinicians to meticulously monitor the clinical symptoms exhibited by patients. For those presenting with symptoms, prompt diagnosis and appropriate symptomatic management are essential. Additionally, regular thyroid function testing is recommended for high-risk patients, and we advocate for the assessment of baseline levels of thyroid peroxidase antibodies (TPOAb) and thyroglobulin antibodies (TGAb) prior to initiating ICI treatment.

## 1 Introduction

Immune checkpoint inhibitors (ICIs), targeting the programmed cell death 1 receptor (PD-1)/programmed cell death ligand 1 (PD-L1) and cytotoxic T-lymphocyte-associated protein 4 (CTLA-4) pathways, have transformed cancer treatment. These antibodies are increasingly used alone or with other therapies for metastatic and locally advanced cancers, particularly lung cancer (1, 2). However, they can cause a variety of adverse events (AE), some life-threatening, if not quickly identified and managed (3).

Thyroid disorders are common immune-related adverse events (irAEs), but severe cases (grade 3 or higher) are rare (4). Sintilimab, a PD1-directed IgG4 monoclonal antibody, is approved in China for lung, gastric/gastroesophageal adenocarcinomas, esophageal squamous cell carcinoma, and liver cancer (5). Trials combining sintilimab with chemotherapy reported no severe thyroid toxicity (6–9).

We present a case of grade 3 thyroiditis in a patient with non-small cell lung cancer (NSCLC) after one cycle of sintilimab monotherapy, following the CARE reporting checklist (available at http://dx.doi.org/10.21037/apm-20-2449).

## 2 Case presentation

**Figure 1:**
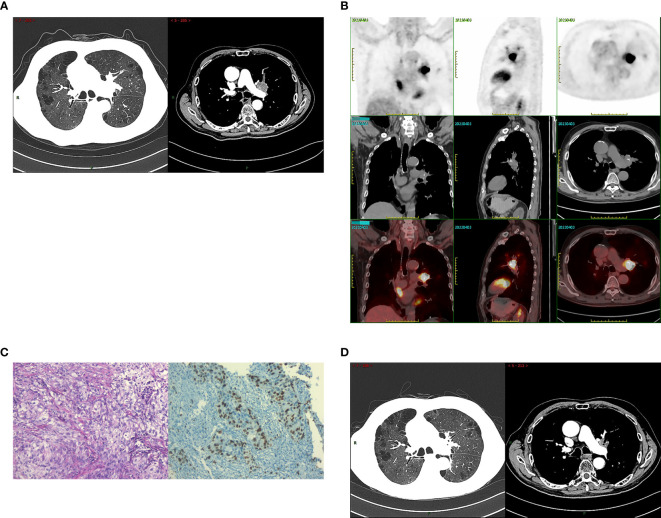
The radiological data at the initial diagnosis of patients and the evaluation of radiotherapy efficacy. **(A)** The patient’s chest CT on March 29, 2023: a space-occupying lesion in the left upper lobe. **(B)** The patient’s PET/CT on April 3, 2023: increased FDG uptake in the lesion, with obstructive pneumonia in the left upper lobe. No hilar or mediastinal lymphadenopathy was observed. **(C)** Tumor tissues were observed under light microscope (HE*200) and the immunohistochemical results: Ki67 index of about 40%(IHC*200). **(D)** The patient’s chest CT on June 29, 2023: the tumor showed a marked decrease in size. Radiotherapy efficacy was assessed as a partial response (PR).

A 76-year-old male patient, who is an active smoker, underwent a thoracoscopic right lower lobectomy with lymph node dissection on July 15,2020. Postoperative pathological analysis confirmed a diagnosis of lung adenocarcinoma with pathological staging IB2, pT1bN0M0. During a follow-up examination on March 29, 2023, a chest computed tomography (CT) scan identified a space-occupying lesion in the left upper lobe (**Figure 1A**). A subsequent 18-FDG positron emission tomography/computed tomography (PET/CT) scan indicated increased FDG uptake in the lesion, with obstructive pneumonia in the left upper lobe. No hilar or mediastinal lymphadenopathy was observed (**Figure 1B**). A bronchoscopic biopsy on April 4, 2023, indicated adenocarcinoma, with immunohistochemistry showing cytokeratin (CK)7(+), CK5/6 (–), thyroid transcription factor (TTF)-1(+), NapsinA (–), p40 (–),and a Ki67 index of about 40% (**Figure 1C**). Imaging and pathology confirmed stage IIIA adenocarcinoma in the left upper lung (cT4N0M0). Next-generation sequencing (NGS) foundno driver gene mutations for targeted therapy. PD-L1 expression analysis using the 22C3 antibody (DAKO) showed a TPS of 55%.

The patient received radical radiotherapy targeting the left lung tumor region, with a total dose of 66 Gy administered in 33 fractions. Based on the Response Evaluation Criteria in Solid Tumors (RECIST) version 1.1, the treatment efficacy was assessed as a partial response (PR) (**Figure 1D**). Immunotherapy with sintilimab at a dosage of 200 mg every three weeks commenced on June 20, 2023.

**Figure 2:**
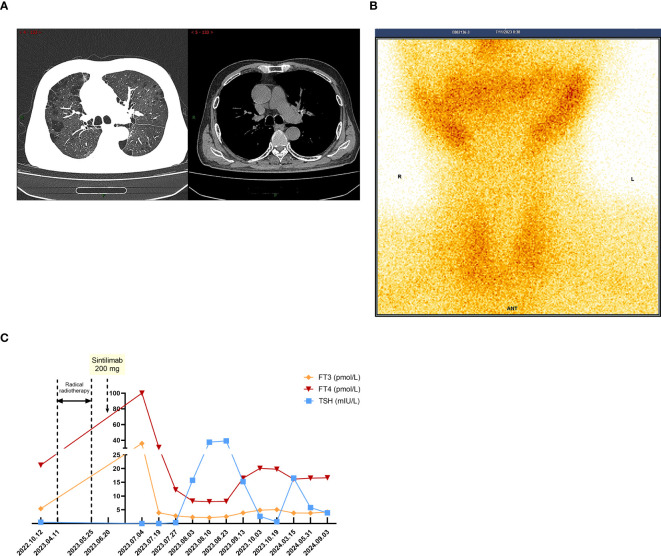
When TD-irAEs occur, the imaging and laboratory examinations of the patient. **(A)** The patient’s chest CT on July 3, 2023: no interstitial lung inflammation. **(B)** The patient’s SPECT/CT on July 11, 2023: thyroid technetium uptake function significantly reduced. **(C)** Time-course changes in the levels of TSH and FT3/FT4 in this case.

**Figure 3:**
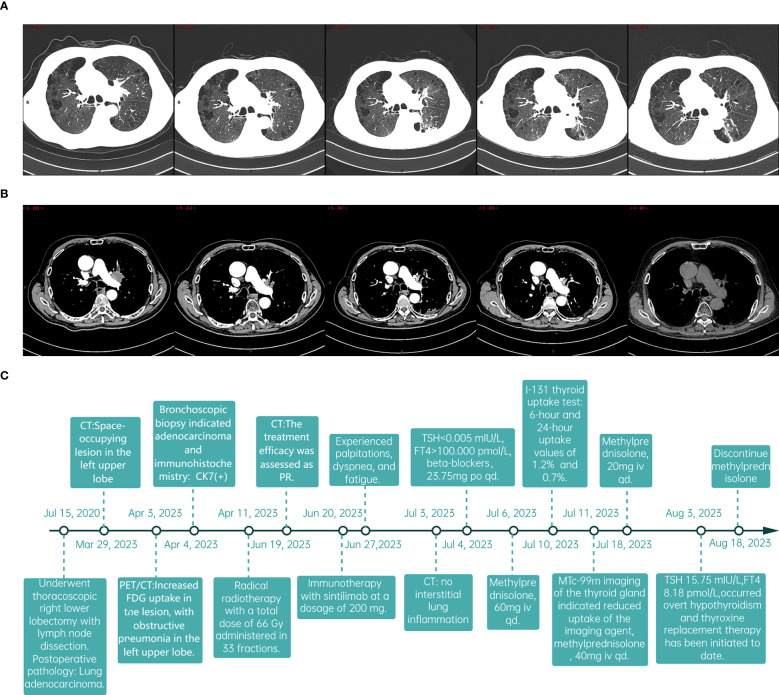
The follow-up data of patients and the timeline. **(A, B)** Sequentially from left to right, the dates indicate the patient’s CT scans: March 29, 2023; June 19, 2023; September 13, 2023; January 25, 2024; and September 3, 2024. **(C)** The timeline with relevant data from the episode of care.

The patient was admitted on July 3, 2023, with a week-long history of palpitations, dyspnea, fatigue, and a 6kg weight loss, worsening over the last day. Tachycardia was observed during the physical exam. A chest CT scan showed no interstitial lung inflammation (**Figure 2A**), ruling out radiation and immune checkpoint inhibitor-related pneumonitis. Myocardial enzymes, B-type natriuretic peptide, and creatine kinase levels were within normal ranges, and myocarditis related to ICIs was ruled out. Serum thyroid function tests revealed a thyroid-stimulating hormone (TSH) level of less than 0.005mIU/L (reference range 0.27-4.2mIU/L) and free thyroxine (FT4) values exceeding 100.000pmol/L (reference value 12.0-22.0 pmol/L). Additionally, thyroglobulin antibodies (TGAb) were measured at 167.5IU/ml (reference value 0.0-115.0 IU/ml) and thyroid peroxidase antibodies (TPOAb) were greater than 600 IU/ml (reference value 0.0-34.0 IU/ml). Thyroid ultrasound demonstrated diffuse lesions within the thyroid. The Iodine-131(I-131) thyroid uptake test showed 6-hour and 24-hour uptake values of 1.2% (reference value 7-40%) and 0.7% (reference value 17.0-60.0%), respectively. Furthermore, Technetium-99m (Tc-99m) imaging of the thyroid gland indicated reduced uptake of the imaging agent (**Figure 2B**). The results of both tests indicated that the probable diagnosis for the patient was subacute thyroiditis. Considering the clinical and laboratory context, along with the patient’s prior history of ICI therapy, the diagnosis of ICI-induced thyroiditis was established, initially manifesting as thyroiditis (hashitoxicosis) due to the release of thyroid hormones from the inflamed thyroid gland. According to the Common Terminology Criteria for Adverse Events (CTCAE) Version 5.0, the thyroiditis was classified as grade 3. Formulate a treatment plan in accordance with the NCCN guidelines. Following a three-day course of beta-blockers and supportive care, the patient’s symptoms did not resolve. However, symptom relief was achieved after administering 60 mg of intravenous methylprednisolone daily for five days, followed by gradual tapering and discontinuation by day 43. Serum thyroid function tests performed on August 3, 2023, indicated a TSH level of 15.75 mIU/L and an FT4 level of 8.18 pmol/L. The variations in serum TSH and FT4/FT3 levels are depicted in **Figure 2C**. The patient subsequently developed overt hypothyroidism and commenced thyroxine replacement therapy. Following this, the patient did not undergo any antitumor treatment. At the most recent follow-up in September 2024, no disease progression was observed. CT imaging conducted at various time points is presented in **Figures 3A, B**. The timeline with relevant data from the episode of care is presented in **Figure 3C**.

## 3 Discussion

Previous clinical studies have shown that thyroid dysfunction (TD) is among the most common irAE, with most cases being asymptomatic or mild (grade 1 or 2) (10, 11). The two most common patterns of ICI-related TD are thyroiditis followed by hypothyroidism with an incidence rate of 2-15% (12), and isolated hypothyroidism. Graves’ disease and thyroid eye disease are rare occurrences (13). Thyroiditis typically manifests relatively early, within 3 to 6 weeks after the initiation of treatment (14). In this patient, the clinical presentation was characterized by early onset, occurring one week following the initiation of sintilimab therapy, and severe manifestations. Prompt and accurate differential diagnosis was crucial to prevent the development of a thyroid crisis. The differential diagnosis for ICI-related thyroiditis in this patient was extensive. Initially, disease progression needed to be excluded. Subsequently, it was necessary to rule out radiation pneumonitis. Additionally, ICI-related myocarditis and myositis had to be considered and excluded. Imaging and blood tests ruled out the initial diagnoses. The patient’s thyroid wasn’t in the radiation area, and only sintilimab was used for treatment, excluding radioactive and drug-induced thyroiditis. Hashimoto’s thyroiditis and Graves’ disease were also excluded via the I-131 thyroid uptake test.

The destruction of the thyroid gland is recognized as the main mechanism behind ICI-related thyroiditis (13, 15), though the exact process remains unclear and may involve multiple factors. Experiments show that cytotoxic memory CD4+ T cells, activated by anti-PD-1 antibodies, are crucial in TD-irAEs development (16). Additionally, nivolumab may induce TD-irAEs by promoting IFN-γ secretion from thyroid cells, attracting CD8+ T cells, and increasing their lethality (17).

However, besides the mechanism of occurrence, the risk factors contributing to its occurrence also remain uncertain. ICI-related thyroiditis is associated with the specific type of ICIs and the combinations of treatments employed. Research indicated that combination therapy was associated with a higher incidence of adverse effects compared to monotherapy (4). The administration of combined PD-1/CTLA-4 therapy had been shown to result in thyroid dysfunction (TD) in approximately 15-20% of patients. In contrast, TD was observed in about 10% of patients receiving anti-PD-1/PD-L1 monotherapy and in 5% of those treated with ipilimumab monotherapy (4, 18). Moreover, the probability of developing hyperthyroidism was significantly greater with the use of PD-1 inhibitors compared to PD-L1 inhibitors (4). We have systematically gathered data on the incidence rates of hyperthyroidism observed in clinical trials for lung cancer involving commonly utilized PD-1 and PD-L1 inhibitors as presented in **Table 1**. The incidence rate for PD-1 inhibitors varies approximately from 0% to 11%, whereas for PD-L1 inhibitors, it ranges from approximately 1.2% to 16%. Notably, within the PD-1 inhibitor category, the incidence rates of hyperthyroidism differ among specific drugs. Patients receiving pembrolizumab demonstrated a higher incidence of hyperthyroidism, aligning with the findings of a meta-analysis conducted by Barroso-Sousa R and colleagues (4). Consistent with other PD-1 inhibitors, the occurrence of ICI-related hypothyroidism was more prevalent than hyperthyroidism, with the majority of cases being asymptomatic or mild (12, 54). Notably, in previous clinical trials (ORIENT 03, ORIENT 11, ORIENT 12, ORIENT 15, ORIENT 16, ORIENT 32, CONTINUUM, NCT04304209), no grade 3 or 4 thyroid dysfunction immune-related adverse events (TD-irAEs) were reported. In the ORIENT 31 study, the combination of sintilimab, the bevacizumab biosimilar IBI305, and chemotherapy (pemetrexed and cisplatin) was found to significantly enhance progression-free survival (PFS) in patients. However, this regimen was also associated with a higher incidence of grade III or higher TD-irAEs (**Table 2**).TD has been observed with antiangiogenesis treatments, especially with tyrosine kinase inhibitors (TKIs) like sunitinib, apatinib, and sorafenib (58, 60–64). One study noted a higher risk of immune checkpoint inhibitor (ICI)-induced TD after previous TKI treatment (59). Therefore, patients should have their thyroid function closely monitored when on combined antiangiogenic and anti-PD1/PD-L1 therapies.

**Table 1:** Incidence rates of all grades and grade≥3 TD-irAEs in phase III clinical trials for NSCLC.

	ICI regimen	Study	Hypothyroidism		Hyperthyroidism	
			All Grades	≥3 Grades	All Grades	≥3 Grades
PD-1 inhibitors	Pembrolizumab	KEYNOTE-024 (19)	9.1%	0.0%	7.8%	0.0%
		KEYNOTE-042 (20)	12.0%	<1%	6.0%	<1%
		KEYNOTE-091 (21)	21.0%	<1%	11.0%	<1%
		KEYNOTE-407 (22)	7.9%	0.4%	7.2%	0.4%
		KEYNOTE-598 (23)	12.1%	0.4%	6.0%	0.0%
		KEYNOTE-671 (24)	13.8%	0.0%	6.6%	0.0%
		KEYNOTE-789 (25)	5.7%	0.0%	5.3%	0.0%
	Nivolumab	CheckMate 017 (26)	5.0%	0.0%	0.0%	0.0%
		CheckMate 057 (27)	19.0%	0.0%	4.0%	0.0%
		CheckMate 078 (28)	4.0%	0.0%	3.0%	0.0%
		CheckMate 722 (29)	2.8%	0.7%	1.4%	0.0%
		CheckMate 816 (30)	2.3%	0.0%	4.0%	0.0%
		CheckMate 77T (31)	11.0%	0.0%	4.8%	0.4%
	Serplulimab	ASTRUM-005 (32)	11.6%	0.3%	9.0%	0.0%
	Sintilimab	ORIENT 03 (8)	14.6%	0.0%	7.6%	0.0%
		ORIENT 11 (33)	7.1%	0.0%	4.5%	0.0%
		ORIENT 12 (6)	10.1%	0.0%	2.8%	0.0%
		ORIENT 31 (34)	10.0%	0.0%	8.0%	1.0%
	Toripalimab	CHOICE-01 (35)	11.4%	0.3%	7.8%	0.0%
		Neotorch (36)	14.4%	0.0%	10.4%	0.0%
	Cemiplimab	EMPOWER-Lung1 (37)	6.0%	0.0%	4.0%	0.0%
		EMPOWER-Lung3 (38)	7.7%	0.3%	5.1%	0.0%
	Camrelizumab	CameL (39)	10.2%	0.5%	4.4%	0.0%
		CameL-sq (40)	11.4%	0.0%	4.7%	0.0%
	Tislelizumab	RATIONALE-304 (41)	8.6%	0.0%	2.7%	0.0%
		RATIONALE-307 (42)	12.2%	0.0%	2.6%	0.0%
		RATIONALE-315 (43)	15.0%	1.0%	7.0%	<1%
PD-L1 inhibitors	Durvalumab	AEGEAN (44)	9.2%	0.0%	1.7%	0.0%
		PACIFIC (45)	9.3%	0.2%	2.7%	0.0%
		POSEIDON (46)	6.0%	0.0%	1.2%	0.3%
	Atezolizumab	IMpower010 (47)	17.0%	0.0%	7.0%	<1%
		IMpower110 (48)	9.4%	0.0%	4.5%	0.0%
		IMpower130 (49)	14.8%	0.6%	4.9%	0.2%
		IMpower150 (50)	12.7%	0.3%	4.1%	0.3%
		CONTACT-01 (51)	17.8%	0.0%	1.6%	0.0%
	Sugemalimab	GEMSTONE-301 (52)	19.2%	<1%	16.0%	0.0%
		GEMSTONE-302 (53)	11.0%	0.0%	7.0%	0.0%

**Table 2:** Incidence rates of all grades and grade≥3 TD-irAEs in phase III clinical trials of sintilimab.

	Hyperthyroidism		Hypothyroidism	
	All grades	Grade≥3	All grades	Grade ≥3
ORIENT 03 (8)	7.6%	0%	14.6%	0%
ORIENT 11 (33)	4.5%	0%	7.1%	0%
ORIENT 12 (6)	2.8%	0%	10.1%	0%
ORIENT 15 (7)	5.8%	0%	12.5%	0%
ORIENT 16 (9)	6.1%	0%	13.7%	0%
ORIENT 31 (34)	8.0%	1%	10.0%	0%
ORIENT 32 (55)	0.0%	0%	14.0%	0%
CONTINUUM (56)	19.0%	0%	28.0%	0%
NCT04304209 (57)	6.0%	0%	6.0%	0%

Patients with a history of autoimmune diseases may be at an increased risk for immune-related adverse events, such as thyroiditis (65). Studies indicate that TD-irAEs may arise from the activation of pre-existing subclinical thyroiditis prior to immune checkpoint inhibitor (ICI) therapy (13, 66, 67). In this case, the patient showed elevated TGAb and TPOAb levels in 2022 without symptoms, suggesting pre-existing latent chronic autoimmune thyroiditis. Previous research has not identified elevated TPOAb levels as a causative factor for TD-irAEs (66, 68). However, a recent prospective study stratified the risk of TD-irAEs induced by anti-PD-1 antibodies based on the presence of TGAb and TPOAb prior to treatment. The study found that patients who were positive for TGAb alone, as well as those positive for both TGAb and TPOAb at baseline, exhibited the highest risk, with an increased incidence of thyroiditis and hypothyroidism observed in both groups (69). These findings indicate that TGAb and TPOAb levels might predict the risk of TD-irAEs, and further study is needed. Patients with high TSH levels were also more prone to TD-irAEs (70). When conducting regular thyroid function tests, stratify based on baseline TPOAb and TGAb levels. For patients without abnormalities, follow guidelines to test TSH and FT4 every 4-6 weeks during ICI treatment and every 6-12 months afterward (71). For patients with elevated TPOAb and TGAb, test thyroid indicators before each medication dose and monitor for symptoms like palpitations, tremors, and weight loss during ICI treatment. Other factors like cancer subtype, sex, age (4, 11, 72, 73), and certain genetic predispositions, such as specific HLA haplotypes (73–76), might also influence the risk of ICI-induced thyroiditis.

Previous comprehensive meta-analyses examining PD-L1 expression in patients with NSCLC and its prognostic implications have demonstrated that elevated tumor PD-L1 expression is associated with reduced survival durations (77–79). Concurrently, other studies have suggested that increased PD-L1 expression in tumor tissues correlates with improved therapeutic outcomes when treated with checkpoint inhibitor therapy (20). Furthermore, some research has indicated that high levels of PD-L1 expression may increase the risk of irAEs (80), and another study has identified a link between the occurrence of checkpoint inhibitor pneumonitis (CIP) and elevated PD-L1 expression (81).In a study focusing on immune-related adverse events in patients with gastrointestinal malignancies, it was found that patients with grade 3-5 irAEs exhibited significantly higher serum PD-L1 levels compared to those with grade 0-2 irAEs. Additionally, an increase in serum PD-L1 levels was associated with the occurrence of rash (82). However, there are currently no studies that definitively clarify the relationship between PD-L1 levels and the severity of TD-irAEs. Further large-scale clinical investigations are warranted to elucidate these potential relationships.

We recommend an endocrine consultation to develop a treatment plan. For thyrotoxicosis symptoms like tachycardia and tremor, use beta-blockers (83). For grade 3 and 4 irAEs, start high-dose glucocorticoids promptly, tapering them as the condition improves or reaches CTCAE grade 1 or lower (84). Thyrotoxicosis often leads to hypothyroidism, so repeat thyroid tests in 4 to 6 weeks. If TSH is over 10 mIU/L, begin thyroid supplementation (15).

## 4 Conclusion

In summary, we report a case of grade 3 thyroiditis in a patient with NSCLC following a single cycle of sintilimab monotherapy. While TD-irAEs are not uncommon, the underlying mechanisms and predictive biomarkers associated with these events remain insufficiently understood. Therefore, it is crucial for clinicians to meticulously monitor the clinical symptoms exhibited by patients. For those presenting with symptoms, prompt diagnosis and appropriate symptomatic management are essential. Additionally, regular thyroid function testing is recommended for high-risk patients, and we advocate for the assessment of baseline levels of TPOAb and TGAb prior to initiating immune checkpoint inhibitor treatment.

## Data Availability

The original contributions presented in the study are included in the article/supplementary material. Further inquiries can be directed to the corresponding author.
